# Improving early gastric cancer detection in upper gastrointestinal series adding digital tomosynthesis (DTS): a preliminary study

**DOI:** 10.1007/s11604-025-01767-9

**Published:** 2025-04-11

**Authors:** Masaki Tachi, Koji Sohara, Shinichiro Kumita

**Affiliations:** Department of Radiology, Nippon Medical School, 1-1-5, Sendagi, Bunkyo-Ku, Tokyo, Japan

**Keywords:** Digital tomosynthesis, Upper gastrointestinal series, Upper gastrointestinal radiography, Early gastric cancer, Double-contrast method, Iterative reconstruction

## Abstract

The aim of this study is to evaluate the effectiveness of digital tomosynthesis (DTS) for improving the detection rate of early gastric cancer (EGC) in upper gastrointestinal (UGI) radiography. We retrospectively analyzed 57 patients with 62 pathologically confirmed EGC lesions. Imaging accuracy was compared between conventional double-contrast UGI and UGI with DTS, using endoscopy as the gold standard.

In experienced readers, the detection rate improved from 71.0% with conventional radiography to 83.9% with DTS. For less experienced readers, the detection rate significantly increased from 53.4% to 79.0%. For lesions < 2 cm, DTS significantly improved detection only for less experienced readers (p < 0.05). For 2–3 cm lesions, both readers showed significant improvement (p < 0.05 and p < 0.01, respectively). No significant difference was found for lesions ≥ 3 cm. DTS also significantly improved detection for non-ulcerated lesions, particularly those with elevated structures, in the less experienced reader (p < 0.05).

AUC analysis showed a slight improvement for experienced readers (0.86 → 0.98, p = 0.0604), while less experienced readers demonstrated significant improvement (0.76 → 0.96, *p* < 0.01). Inter-reader agreement for conventional radiography was 0.71, which improved to 0.90 with DTS. The total combined radiation dose for UGI and DTS was 4.7–5.3 ± 0.5 mSv, within acceptable limits.

UGI with DTS significantly enhances EGC detection, particularly for lesions 2–3 cm and non-ulcerated elevated lesions, improving diagnostic accuracy and consistency, especially for less experienced radiologists. Given its minimal additional examination time (143 s) and cost-effectiveness compared to EGD, DTS may serve as a practical supplementary tool for gastric cancer screening and preoperative evaluation.

## Introduction

Gastric cancer arises when cells in the mucosal lining of the stomach undergo uncontrolled proliferation due to defects in the mismatch repair system, leading to disordered growth of mismatch lesions [1]. The incidence of gastric cancer in Japan ranks third among all malignant tumors (combined for both genders). By gender, it ranks fourth in men, following prostate, colorectal, and lung cancers, and fourth in women, following breast, colorectal, and lung cancers. The incidence is more than twice as high in men as in women, with a sharp increase starting in the late 50 s and continuing to rise through the late 80 s [2, 3]. Early gastric cancer refers to shallow tumors confined to the mucosa or submucosa, where metastasis is rarely observed. However, once the cancer extends into the muscularis or beyond, it progresses to advanced gastric cancer, significantly raising the risk of metastasis and postoperative recurrence. Early detection and treatment are thus essential [4]. Although the incidence of gastric cancer has been gradually declining over the years due to the discovery of Helicobacter pylori (HP) and the establishment of eradication methods, it remains prevalent in Japan and East Asia. Delayed detection continues to result in significant costs for treatment and additional examinations each year [5]. The primary diagnostic methods for gastric cancer include upper gastrointestinal (UGI) radiography using contrast agents such as barium and an X-ray TV system, endoscopy (with biopsy and ultrasound), and serum/biochemical testing, including tumor markers. In recent years, genetic diagnostics have also been increasingly researched [6]. In Western countries, the incidence of gastric cancer is lower compared to Japan and other parts of Asia, resulting in the absence of widespread screening programs and relatively limited perspectives on this examination method [7]. In contrast, in Western countries, where the prevalence of gastric cancer is lower compared to Japan and other Asian regions, widespread screening programs are not commonly implemented, and research on this method is relatively limited [8]. In Japan, before the advent of endoscopy, contrast radiography was virtually the sole imaging modality for the digestive tract. The high incidence of gastric cancer further contributed to the development of the double-contrast method, establishing UGI contrast radiography, alongside chest radiography, as the cornerstone of health screening programs. Even today, in health checkups, this examination remains unchanged as a key component due to its simplicity and cost-effectiveness. While its use in hospitals has declined, UGI contrast radiography continues to play a central role in health screening programs alongside chest radiography [9]. Although interest in health has increased, factors such as the shortage of endoscopists and advancements in diagnostic equipment have paradoxically led to a decline in the number of radiologists specializing in gastrointestinal contrast imaging. This trend could increase the workload of experienced radiologists, lower detection rates in screening programs, and contribute to a resurgence of advanced gastric cancer, thereby raising healthcare costs and posing a societal problem. While endoscopy allows for direct visualization of the gastric wall and the option of biopsy, UGI contrast radiography provides only indirect observation and historically has had lower diagnostic accuracy. However, with advancements in technology, such as digital imaging, flat-panel detectors, and improved image processing, highly detailed images can now be obtained compared to traditional film-based systems [10]. Previously, detailed assessment of the mucosal surface was challenging, but with adequate expertise, even minute lesions can now be detected.

**Fig. 1:**
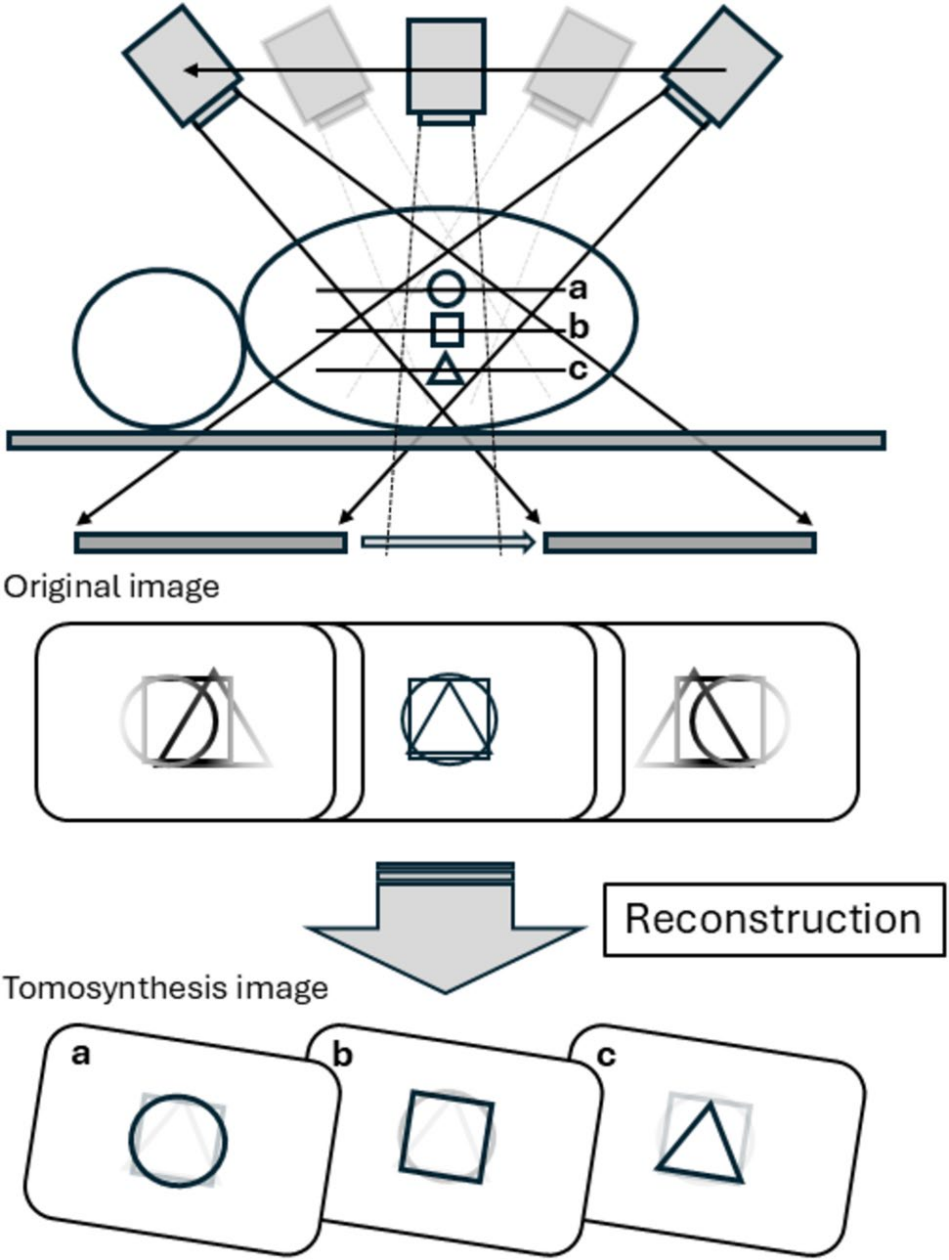
Schematic diagram of parallel section tomography operation in tomosynthesis. **a**-**c** in the diagram represent the differences in depiction for each cross-section, based on the position of the tomographic plane and the tomographic images reconstructed by the workstation. The original image is also considered useful for diagnosis, as the subject can be observed from multiple angles

In addition, modern X-ray equipment is equipped with cone-beam technology, enabling cone-shaped imaging from a fixed point (circular tomographic scanning). This allows for continuous multi-angle observation and the reconstruction of virtual tomographic images (tomosynthesis) [11]. The use of tomosynthesis has been widely studied and clinically adopted in fields such as breast and orthopedic surgery [12, 13]. In gastrointestinal radiography, angled imaging is frequently performed [14], and by continuously varying the imaging angle (parallel planar tomography) and reconstructing the data, virtual tomographic images of the digestive tract can be generated [15] (Fig. 1). When combined with the double-contrast technique used in UGI radiography, this approach can generate multi-directional double-contrast images and semi-transparent tomographic images of the mucosal surface. This has the potential to enhance the detection of small lesions, such as those seen in early-stage gastric cancer, and reduce diagnostic discrepancies between radiologists with different levels of experience.

The aim of this study is to demonstrate that the use of the double-contrast technique for visualizing the gastric mucosa, in combination with tomosynthesis and iterative reconstruction methods, can improve the depiction and recognition of early gastric cancer lesions that may not be detectable with conventional UGI contrast radiography alone. we have examined the identification of lesions as a preliminary step to detailed clinical diagnostic steps and explore potential role for health checkup in gastric cancer screening.

## Materials and methods

The subjects included 57 cases with 62 lesions, which were referred to either the Department of Gastroenterological Surgery or the Department of Gastroenterology at this hospital between April 2018 and March 2024 for early gastric cancer. All patients had already received a pathological diagnosis through endoscopic biopsy, and gastric radiography was requested as part of the preoperative examination. The cohort consisted of 35 males and 22 females, with ages ranging from 46 to 83 years (mean age ± standard deviation: 68 ± 11.1 years). The details of these cases are provided in a separate document (Table 1).

**Table 1:**
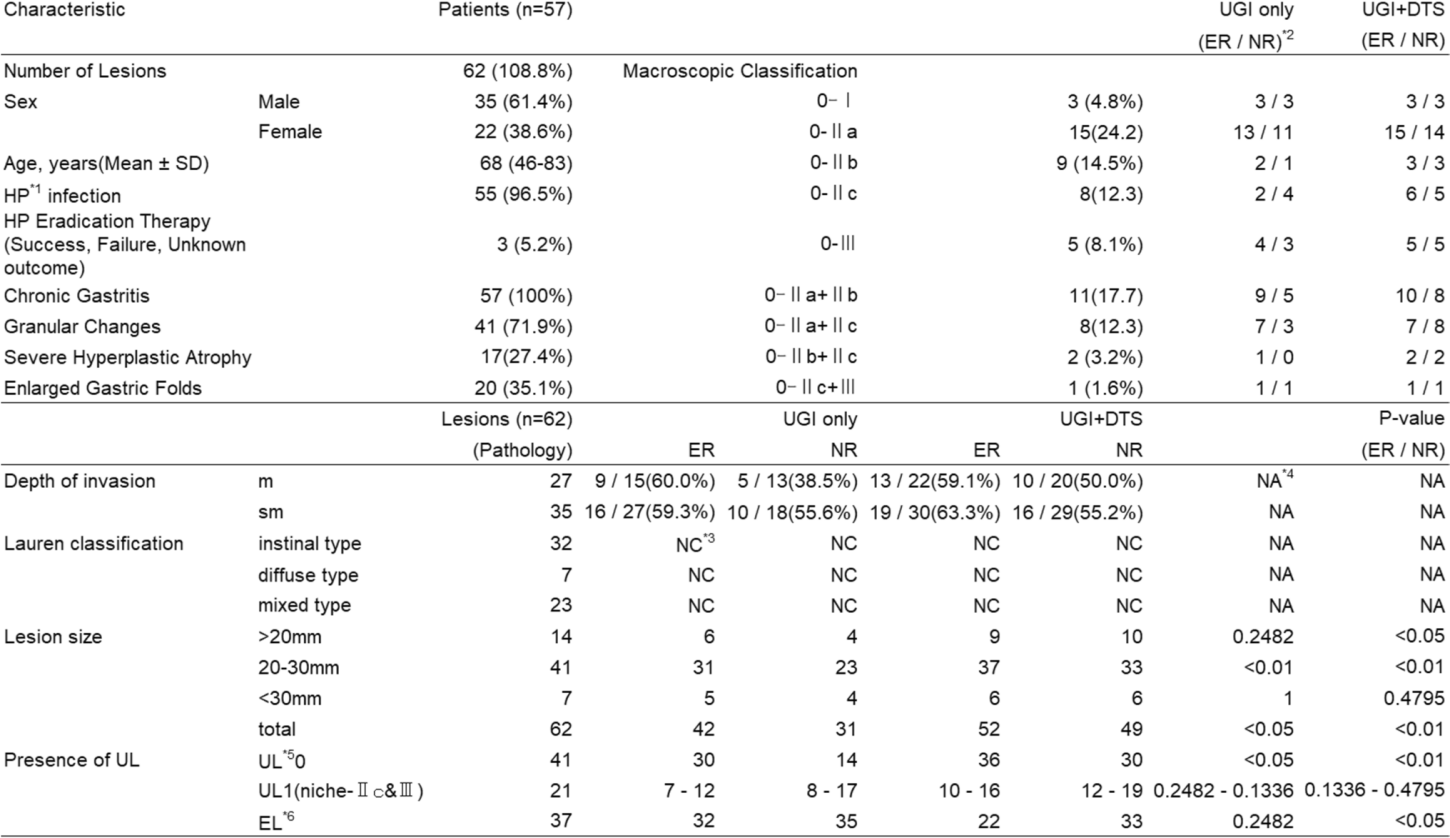
Demographic and Clinical Characteristics of patients in this study

^*1^*Helicobacter pylori*, ^*2^expert reader/novice reader, ^*3^not classified, ^*4^not applicable, ^*5^ulcer, ^*6^elevated lesions

The imaging system used for this study was the SONIALVISION G4/Safire II X-ray TV system manufactured by Shimadzu Corporation. Both conventional gastric radiography and reconstructed tomographic imaging of the gastric mucosa, called tomosynthesis, were performed to depict early gastric cancer lesions.

For the imaging procedure, a barium solution (150 g of barium dissolved in 150–180 ml of water) was ingested, followed by two rotations of the patient on the fluoroscopic table. Imaging was conducted in the supine position (focusing on the body and antrum-pylorus regions) and the Schatzki position (focusing on the fundus and upper body regions). The parallel planar tomographic scan was directed from the cranial to caudal axis, centering on the gastric focus. This imaging method was carried out in addition to conventional gastric radiography techniques. The second tomosynthesis imaging was added because, in the supine position, the contrast agent tends to accumulate in the fundus and upper body, making it difficult to observe the mucosal surface. Thus, Schatzki position imaging was performed to supplement the two-directional double-contrast images of the entire stomach.

The images acquired through parallel planar tomographic scanning (tomosynthesis raw images) were reconstructed into tomographic images using iterative reconstruction on the Shimadzu workstation attached to the X-ray TV system. The reconstructed tomographic images and conventional gastric radiography images were interpreted by two radiologists, one with over 15 years of experience and the other with less than 2 years of experience.

The iterative reconstruction method reduces image blurring caused by out-of-focus structures, which is an inherent issue in traditional filtered back projection (FBP) methods. This is achieved by generating projection data from initial tomographic images and iteratively reducing errors between the actual data and the projections, resulting in clearer images [16].

Conventional gastric radiography included images taken in upright, supine, anterior supine, first and second oblique, Schatzki position, and compression views. The study first evaluated the diagnostic differences between using only conventional gastric radiography images and using both parallel planar tomographic and tomosynthesis images, assessing the impact on lesion depiction.

Three sets of images were used for the comparison: conventional upper gastrointestinal series (UGI), digital tomosynthesis (DTS), and endoscopic images taken prior to this examination (GIF). Specifically, the following analyses were conducted: (1) Sensitivity and specificity of UGI images alone and UGI + DTS images were calculated for each observer, with the GIF images used as the gold standard (set to 100). (2) McNemar's test was used to compare the paired proportions, with a p-value less than 0.05 considered statistically significant. Additionally, lesion characteristics, including tumor type, size, and the presence or absence of ulcers, as determined by pathological examination, were compared with previous reports [17] to assess potential differences in detectability among different imaging modalities. (3) Receiver Operating Characteristic (ROC) curve analysis was performed for each observer, using a 5-point scale (5: definitely depicted, 4: probably depicted, 3: indeterminate, 2: probably not depicted, 1: definitely not depicted) to compare the Area Under the Curve (AUC) scores. (4) Interobserver agreement on lesion depiction between UGI and DTS was assessed using kappa statistics separately. (5) The total radiation dose was measured and compared to conventional gastric radiography and abdominal CT to evaluate the validity of the examination. Statistical analysis was conducted using EZR (Ver. 1.68).

Exclusion criteria included patients under the age of 20 at the start of the study, in consideration of radiation exposure. Pregnant women were also excluded. Additionally, patients who had not undergone endoscopic biopsy or received a pathological diagnosis prior to the study, patients with a history of gastric surgery, or those who had difficulty completing the examination due to communication barriers were also excluded.

Prior to the analysis of the research results, the information regarding the study and the opt-out notice were published on the Clinical Research Center's website at Nippon Medical School Hospital. This ensured that the participants were given the opportunity to refuse participation in the study. Patients or their representatives who expressed a desire to opt out were promptly excluded from the study.

## Results

Among the 57 patients, 5 had two lesions. Fifty-five patients had HP infection, and 3 patients presented with gastric cancer lesions despite having undergone eradication therapy (eradication status unknown). All 57 patients exhibited a baseline of chronic gastritis, with findings of granular changes, enlarged folds, and/or severe hyperplastic atrophy observed in every case (Table 1).

Among the lesions resected by surgery or endoscopic submucosal dissection (ESD), 32 were classified as the intestinal type, 7 as the diffuse type, and 23 as the mixed type based on pathological findings.

Detection Rates (Sensitivity): For the experienced radiologist, the detection rate for early gastric cancer using conventional gastric radiography alone was 71.0%, and when combined with digital tomosynthesis (DTS), it increased to 83.9%. For the less experienced radiologist, the detection rate was 53.4% with conventional radiography alone and improved to 79.0% with the addition of DTS. As all cases examined were positive, the specificity for both radiologists in all cases was 0.

Detection of Early Gastric Cancer Lesions: Among the 62 early gastric cancer lesions, the experienced radiologist was able to detect 42 lesions using conventional gastric radiography alone. With the addition of DTS, 10 more lesions were identified, resulting in a total of 52 lesions detected (McNemar's test, p = 0.04331, at a cautious 5% significance level). The less experienced radiologist detected 31 lesions with conventional radiography alone, and with DTS, an additional 18 lesions were identified, bringing the total to 49 lesions detected (McNemar's test, p = 0.005578, at a cautious 5% significance level).

The depth of invasion was classified as mucosal (m) in 27 lesions and submucosal (sm) in 35 lesions. The diagnostic accuracy ranged from 59 to 63% for experienced readers and from 39 to 56% for less experienced readers, with partial improvement observed following the addition of DTS.

For lesions < 2 cm, statistical significance was not observed for the experienced radiologist (p = 0.2482), but was achieved for the less experienced radiologist (p < 0.05). In 2–3 cm lesions, both radiologists showed significant improvement (p < 0.05 and p < 0.01, respectively), with greater gains for the less experienced reader using DTS. No significance was found for lesions ≥ 3 cm (p = 0.4795), likely due to the small sample size. Ulcer presence showed no significant differences in macroscopic classification or niche detection. However, for non-ulcerated lesions, both radiologists showed significant improvements (p < 0.05 / p < 0.01). When limited to elevated lesions, the less experienced radiologist exhibited a significantly higher detection rate (p < 0.05) (Table 1).

Ulcer presence showed no significant differences in macroscopic classification or niche detection. However, for non-ulcerated lesions, both radiologists showed significant improvements (p < 0.05 / p < 0.01). When limited to elevated lesions, the less experienced radiologist exhibited a significantly higher detection rate (p < 0.05) (Table 1).

**Fig. 2:**
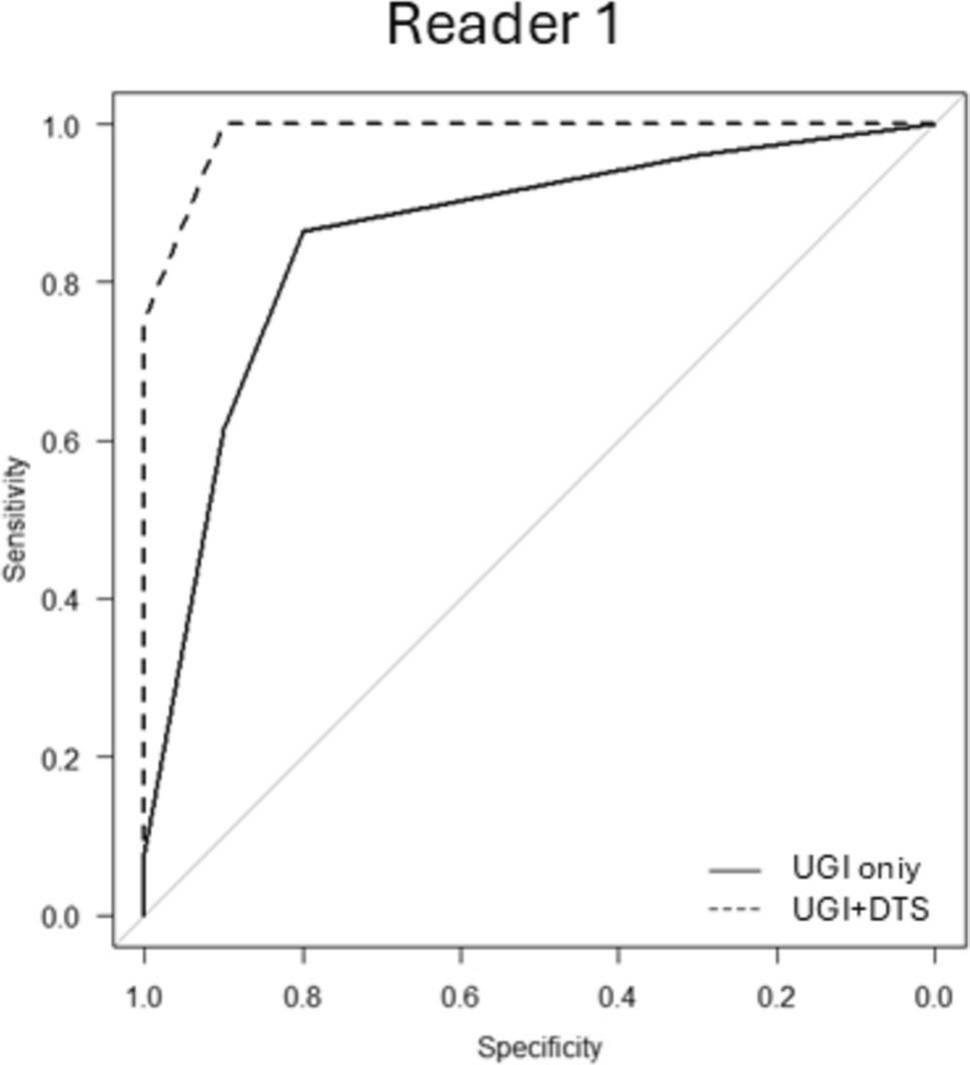
The comparison of ROC curve AUCs between readers with and without DTS (expert reader). For the expert reader, the area under the ROC curve (AUC) showed a slight improvement from 0.86 to 0.98 (p = 0.0604). Although this change did not reach statistical significance, it suggests a clinically meaningful enhancement in diagnostic performance. Even for radiologists with already high diagnostic accuracy, the addition of DTS may provide further confidence and reliability in lesion detection

**Fig. 3:**
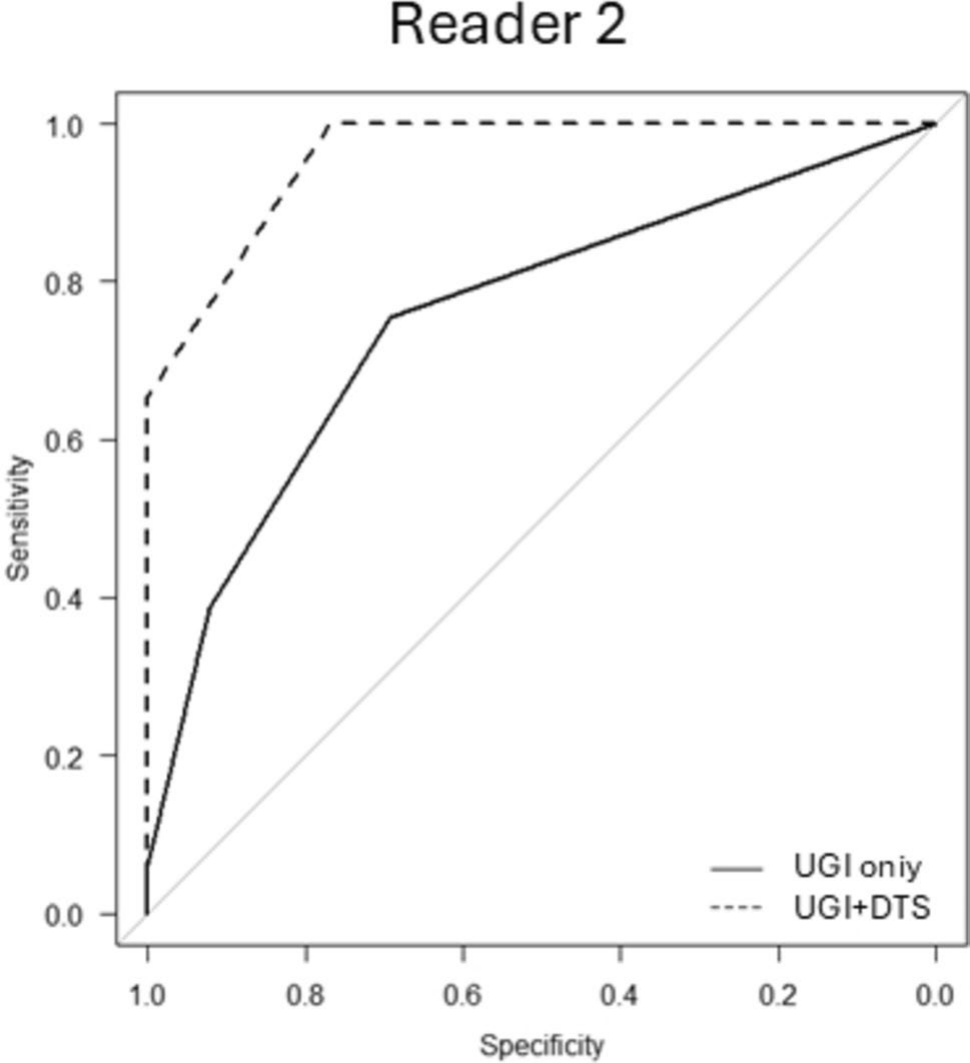
The comparison of ROC curve AUCs between readers with and without DTS (novice reader). For the novice reader, the AUC significantly increased from 0.76 to 0.96 (p = 0.00173). This marked improvement demonstrates that DTS effectively compensates for limited reading experience, greatly enhancing diagnostic accuracy. These findings indicate that DTS serves as a valuable supportive tool, particularly beneficial for novice radiologists or those still in training

Comparison of AUC between Readers: In terms of the Area Under the Curve (AUC) for detecting lesions, a slight improvement was observed for the experienced radiologist, with the AUC increasing from 0.86 to 0.98 (p = 0.0604) (Fig. 2). For the less experienced radiologist, the AUC showed a significant improvement from 0.76 to 0.96 (p = 0.00173) (Fig. 3).

Interobserver Agreement: The kappa statistic for interobserver agreement in detecting lesions using conventional gastric radiography alone was 0.71. With the addition of DTS, the kappa value improved to 0.90 (Confidence Interval = 0.95), indicating a higher level of agreement between the two radiologists.

Radiation Dose: The average radiation dose for the 62 cases using conventional gastric radiography alone was 2510.6 μSv (range: 2178.3–2671.8 μSv, ± 358 μSv). For DTS alone, the average dose was 1248.5 μSv (range: 1037.9–1552.8 μSv, ± 289 μSv). The total combined radiation dose for conventional gastric radiography and DTS was 5041.6 μSv (range: 4686.2–5321.1 μSv, ± 554 μSv).

## Discussion

The current study focused on cases already diagnosed with gastric cancer through pathological examination via endoscopy. Detection rates were calculated using endoscopy as the gold standard (100%), and the AUC reflects detection capability relative to endoscopy.

When comparing UGI alone to UGI + DTS, the sensitivity and specificity increased for all readers after the additional test. Although the p-value for experienced readers did not reach the conventional threshold of significance in McNemar's test (p = 0.0604), a strong trend was observed, and significant improvement was found for less experienced readers (p = 0.00173). In experienced readers, there was a mild improvement in AUC, but since the p-value did not reach statistical significance (typically set at 0.05), the improvement was not considered statistically significant, though the result was near the threshold. The diagnostic accuracy of experienced readers was originally high, and although no significant difference was observed, the results were close to endoscopic findings, suggesting a clinically important improvement. For less experienced readers, the diagnostic accuracy was significantly improved, indicating that the addition of DTS may compensate for the lack of reading skills and may enhance both confidence and ability in diagnosis, even for less experienced individuals.

The evaluation of invasion depth using pathological specimens showed that the classification of mucosal (m) and submucosal (sm) lesions was slightly lower to comparable to previous reports. Although the number of concordant cases increased with the addition of DTS, the overall number of detected lesions also increased, resulting in only partial improvement in diagnostic accuracy. Both overestimation and underestimation were frequently observed, and while certain patterns appeared to exist, it remained difficult to distinguish these classifications strictly, even when compared with endoscopic depth assessment, indicating potential diagnostic limitations. This may be attributed to the nature of UGI, in which barium adheres thinly to the gastric mucosal surface and is visualized indirectly through transmitted radiation as a double-contrast image. Consequently, even with reconstructed tomographic images, structures beneath the mucosal surface are not adequately visualized. Size-based evaluation revealed that for small lesions < 2 cm, significant improvement in detection was observed only for less experienced readers, though the effect size was relatively weak. However, even for experienced readers, some lesions became discernible with DTS, and for lesions ≥ 2 cm, the addition of DTS significantly improved detection rates. Due to the limited sample size of lesions ≥ 3 cm in this study, precise statistical evaluation was not possible. While detection rates are expected to increase with lesion size, UGI alone is also likely to show improved detection, potentially diminishing the statistical significance of DTS addition. At present, the most notable improvement was observed in lesions measuring 2–3 cm. The significance of improved detection for non-ulcerated lesions appears to be influenced by a mix of both elevated and flat lesions, suggesting that DTS enhances lesion visibility particularly when an elevated structure is present.

**Fig. 4:**
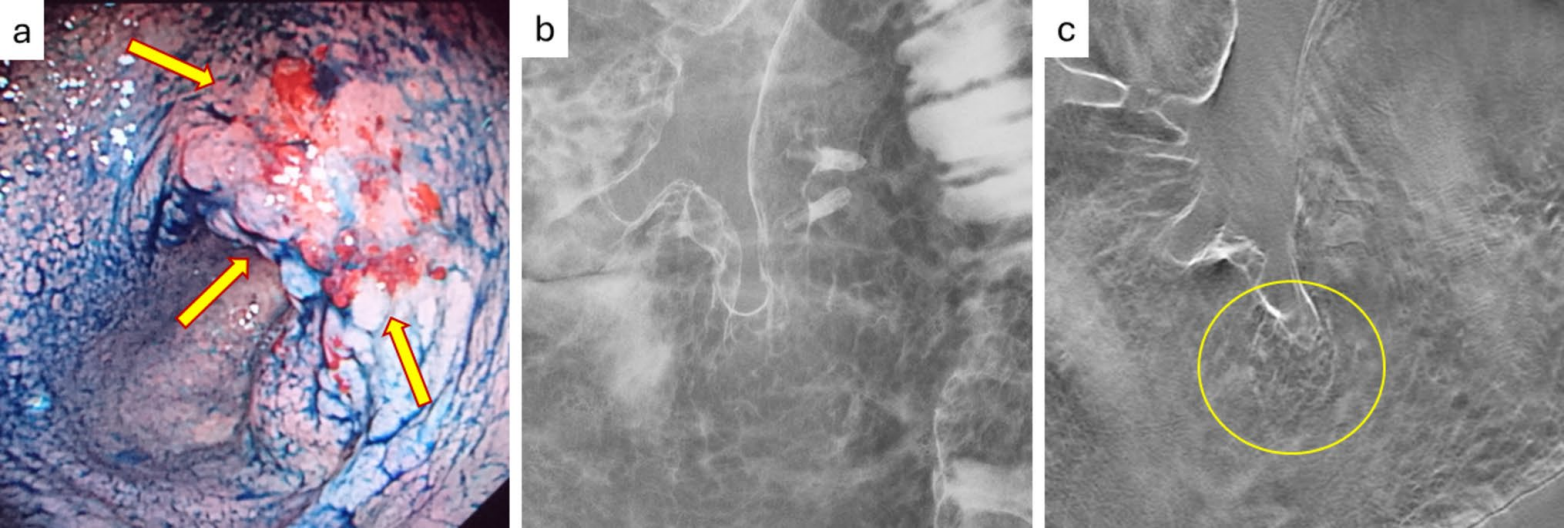
A case of depiction of early gastric cancer 1 (80 yr, male, type IIb + IIc). This figure shows an example of early gastric cancer visualized using different diagnostic methods, where endoscopy **a** clearly depicts the lesion, conventional Upper Gastrointestinal (UGI) radiography **b** fails to show it, highlighting the limitations of using only UGI for detection, and tomosynthesis **c** provides a layered image of the mucosal surface, making it clearly easier to identify the lesion. This comparison demonstrates that combining UGI and tomosynthesis can improve the visibility and detection accuracy of early gastric cancer

**Fig. 5:**
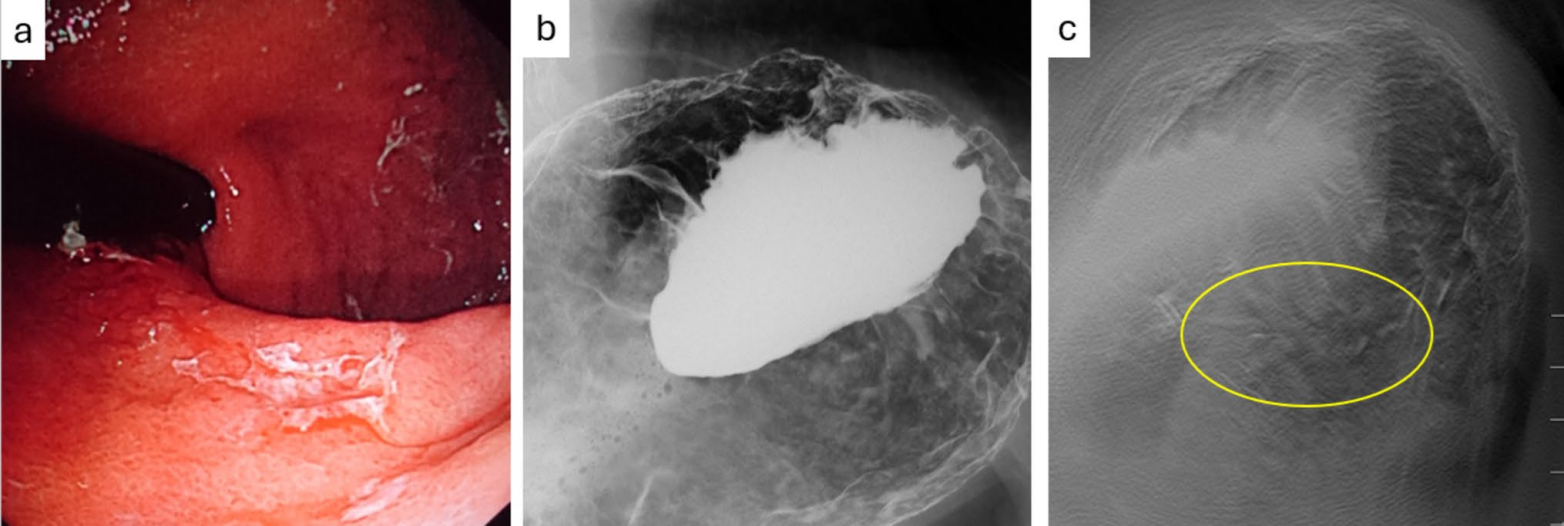
A case of depiction of early gastric cancer 2 (68 yr, male, type IIc). The lesion was located on the anterior wall of the upper gastric body, and due to the horizontal position of the stomach (**a**, EGD), it was difficult to distinguish the lesion with UGI alone as the barium accumulated in the fundus in the supine position (**b**, UGI). However, tomosynthesis allowed clear identification of the lesion, even on the anterior wall, which is generally challenging to visualize (**c**, Tomosynthesis)

The kappa values in conventional UGI examinations indicated relatively consistent diagnoses among readers, though some discrepancies remained. The addition of DTS resulted in a noticeable increase in kappa values, showing that the agreement between readers significantly improved. DTS likely provides more detailed information than conventional 2D images, generating 3D-like images that facilitate the detection of lesions and abnormalities. As a result, diagnostic concordance among readers improved, suggesting a reduction in difficult cases in UGI examinations and an enhancement in diagnostic accuracy. Furthermore, it was demonstrated that even when different physicians read the same patient’s images, they were more likely to reach the same conclusion, indicating that DTS offers more objective information with less reliance on the physician’s experience or skill (improved reproducibility) (Table 1, Figs. 4, 5).

Clinical Implications.

Improving diagnostic accuracy in UGI examinations can enhance the overall diagnostic quality for patients, reducing the risk of unnecessary pre-treatment examinations, treatments, or follow-up tests. This could increase diagnostic consistency between examiners and facilities, potentially contributing to improved patient safety and treatment quality. The significant improvement in inter-reader agreement due to the addition of DTS offers a particular advantage in diagnosing challenging cases and small lesions. This supports the utility and justification of incorporating DTS into medical facilities, though it may not yet become part of the standard protocol for UGI examinations. However, there is a legitimate basis to consider its supplementary use in specific cases (e.g., when gastritis is evident during the examination).

Although the presence or absence of ulcers or structural classifications such as elevation does not necessarily influence the decision for additional testing or treatment, DTS should be actively utilized, even at the discretion of radiological technologists, when an elevated structure is suspected (excluding obvious fundic gland polyps).

In terms of radiation exposure, the average radiation dose for abdominal CT is approximately 5000–10000 μSv (5–10 mSv, with an average of 7.7 mSv) [18, 19]. In comparison, both the standard UGI protocol and DTS have radiation doses lower than the lower threshold for abdominal CT, and even when performing UGI + DTS twice, the total dose approaches the lower end of abdominal CT levels. While abdominal CT provides a wide range of information in a single scan, UGI + DTS is limited to the stomach, and it is difficult to evaluate the gastric mucosa in detail using only MDCT [20]. Therefore, the radiation exposure from UGI + DTS is reasonable compared to CT, and the detail of diagnostic information obtained is also considered reasonable.

In the field of chest screening, imaging technologies and image processing techniques such as Bone Suppression and AI have been developed to increase detection rates of lesions. However, in the upper gastrointestinal region, the wide variety of artifacts, individual variations in gastric morphology, and diverse imaging patterns have hindered progress.

On the other hand, advancements in endoscopic technology have progressed significantly, including the widespread use of transnasal endoscopy, miniaturization of equipment, enhanced imaging endoscopy (such as NBI and BLI), and the development of AI-assisted diagnosis. These innovations suggest the potential for improved detection accuracy of early gastric cancer and reduced variability in diagnostic accuracy among readers. In particular, AI-assisted diagnosis is expected to help bridge the gap in diagnostic accuracy between endoscopists with varying levels of experience. These technological advancements have greatly contributed to the improvement of gastric cancer diagnosis, and further developments are anticipated in the future.

However, the advancement of endoscopic techniques does not render UGI obsolete. Rather, it is essential to utilize each diagnostic modality according to its strengths and ensure their appropriate integration. While endoscopy allows for detailed observation and biopsy, its application to all patients is not practical due to constraints on examination time and medical resources.

In contrast, UGI + DTS serves as a valuable approach within the widely implemented UGI examination, particularly for cases requiring additional evaluation or for secondary screenings aimed at improving diagnostic accuracy and cost-effectiveness. Furthermore, the addition of DTS is beneficial in situations where UGI alone presents diagnostic uncertainties or when immediate access to endoscopy is not feasible.

Although the implementation of DTS requires investment in equipment and infrastructure, the combination of endoscopy and UGI + DTS enables flexible operation tailored to the diagnostic framework of each facility and the characteristics of its patients. Therefore, while evaluating the progress of endoscopic technology, it is crucial to effectively utilize UGI + DTS to establish a screening system that balances accuracy and efficiency.

The advantages of UGI over EGD include, first and foremost, a shorter examination time. The total duration, including preparation, the procedure itself, and post-procedural cleanup, is approximately 5–8 min. Additionally, UGI requires fewer personnel; while EGD typically involves a doctor and two nurses, UGI can be completed by a single radiological technologist without the need for a physician. Moreover, with mobile fluoroscopy-equipped vans, UGI can be performed outside medical facilities, such as in outdoor settings or company premises.Many patients perceive UGI as less physically burdensome compared to EGD. Allergic reactions to barium (Ba) used in UGI are less common and tend to be less severe than the anesthesia-related allergies associated with EGD. Furthermore, UGI imaging provides a clear view of the entire stomach and allows easy visualization of the relative positional relationship between the stomach and lesions.

Clinical Utility of Adding DTS to UGI.

Practical Utility in Preoperative Assessment: DTS enables three-dimensional visualization of the location and extent of lesions, which can function as intraoperative landmarks. Furthermore, DTS supports endoscopic (EGD) findings, contributing to the optimization of treatment plans for patients. By providing detailed information preoperatively, DTS aids in the decision-making process for treatment strategies, alleviating patient burden and ensuring the selection of the most appropriate therapeutic approach.

Complementary Role in Diagnosis: Adding DTS to UGI compensates for potential blind spots in UGI by detecting lesions that might otherwise be missed, thereby achieving diagnostic accuracy comparable to EGD. DTS also significantly enhances diagnostic performance for less experienced readers, improving consistency and reliability across interpreters. These findings underscore the crucial role of DTS in enhancing diagnostic capabilities and reducing variability among readers.

Utility in Health Checkups.

Short Examination Time: The additional time required for DTS is 143 s (116–187 ± 24 s), which is short enough to maintain the overall efficiency of UGI examinations. The majority of this additional time is spent on setup and imaging procedures, with the actual radiation exposure accounting for only 5–10 s. In typical health checkup centers, the capacity per room is usually 25–30 UGI cases or approximately 15 EGD cases per day. Even with the inclusion of a few DTS cases, the operational efficiency of the examination workflow can be maintained.

Cost-Effectiveness: Adding DTS does not result in significant direct cost increases (excluding minor factors such as electricity and time). It preserves the cost structure of UGI while improving diagnostic accuracy. In comparison, EGD typically costs 1.5–2 times more than UGI, highlighting the superior cost-efficiency of UGI with DTS.

These findings demonstrate that UGI with DTS significantly improves diagnostic accuracy under specific conditions. Furthermore, the reduced examination time and superior cost-effectiveness make UGI + DTS a practical option for use in health checkups and preoperative assessments. The integration of DTS into UGI workflows holds great potential for enhancing diagnostic performance and operational efficiency in various clinical and non-clinical settings.

This study is the first to demonstrate that adding supplementary testing to UGI, which has a low detection rate (particularly for early lesions) and is difficult to diagnose, can improve examination efficiency and diagnostic rates. Although the increase in radiation exposure and examination time, though limited, might restrict its use in cost- and turnover-sensitive screening settings, the benefit is considered substantial if the timing for supplementary use can be identified.

UGI itself has a history of over 100 years and remains widely performed in Japan. However, the number of examinations is gradually decreasing due to the influence of diagnostic accuracy and alternative methods, and there has been little perceived innovation in the examination itself. Nonetheless, advancements in imaging equipment and post-imaging correction technologies have enabled the creation of highly detailed images. As a result, many lesions that were previously difficult to visualize with X-rays can now be depicted in much greater detail, though this has not been widely recognized.

Even for inexperienced physicians, 3D reconstructed images derived from sectional images such as CT or MRI are relatively familiar and contribute to better diagnosis. Therefore, it is expected that diagnostic accuracy will improve significantly for readers beyond those involved in this study.

In 2019, prior to the COVID-19 pandemic, approximately 460,000 individuals underwent EGD as part of health checkups or screening programs, compared to 3.87 million individuals who underwent UGI [21]. Meanwhile, the number of registered endoscopy specialists was around 19,000 [22]. Even with a potential increase in the number of endoscopists in the future, it remains challenging to accommodate all gastric screening patients with EGD alone.

According to the Ministry of Health, Labour and Welfare’s Survey of Physicians, Dentists, and Pharmacists Statistics, the number of board-certified radiologists nationwide was 4,780 in 2004, and it has been steadily increasing each year, reaching 7,112 in the 2020 survey—an almost 1.5-fold increase [23].

Incorporating additional technologies to support less experienced readers and strengthening collaboration with radiological technologists offers a realistic approach to maintaining screening capacity while simultaneously improving diagnostic accuracy.

If further improvements in machine performance and the widespread adoption of this testing method are achieved, further developments in UGI screening can be anticipated.

## Limitation

Although we conducted comparisons between early gastric cancer macroscopic classifications and endoscopic diagnoses, UGI, and UGI + DTS, several biases were identified:

- (1) The tumor type was known before the examination.
- (2) Less experienced readers were more likely to be influenced by endoscopic results.
- (3) The statistical power was insufficient due to the variety of classification subtypes (e.g., IIa-c, IIa + IIc) and the limited sample size in this study.

The detection of flat tumors (particularly type 0-IIb) was difficult with both UGI alone and UGI + DTS. In contrast, tumors with prominent elevation, such as type I, or deep depressions, such as type III, were easier to detect and tended to align well with endoscopic results. However, due to these limitations, conducting statistical analyses on these aspects was deemed challenging in this study.

The addition of DTS resulted in an increase in the kappa value to 0.90, with a 95% confidence interval (CI = 0.95), indicating that the results are highly reliable. However, it should be noted that this study was conducted exclusively on cases where early gastric cancer had already been identified. Therefore, it cannot be assumed that such a high level of agreement would be achieved in all cases encountered in routine health screening.

Cost and equipment issues: The introduction of DTS incurs additional costs, and there is a possibility that preparation of the equipment and interpretation time may take longer compared to standard UGI examinations. Therefore, careful consideration of the cost–benefit balance is essential before implementing DTS in clinical practice.

## Conclusion

UGI with DTS demonstrated significant improvements in the detection rate, diagnostic accuracy, and inter-reader agreement for early gastric cancer, particularly among less experienced readers, compared to conventional UGI alone. These enhancements highlight the potential of DTS as a valuable supplementary tool in improving diagnostic performance. While the efficiency and cost-effectiveness of UGI with DTS remain relatively limited, its ability to provide enhanced diagnostic precision within a reasonable radiation dose suggests a promising role in both screening programs and clinical practice. Further studies are warranted to fully evaluate its broader applicability and long-term impact.

## Data Availability

To protect confidentiality and privacy, all raw data related to the study were handled with strict attention to safeguarding participants' information. Measures were taken to prevent leakage, loss, and damage. Data were anonymized at the time of registration, and each case was assigned a unique case number. The correspondence table linking case numbers to participants was stored securely with password protection and was accessible only to designated study collaborators.
